# Quantitative pupillometry and neuron-specific enolase independently predict return of spontaneous circulation following cardiogenic out-of-hospital cardiac arrest: a prospective pilot study

**DOI:** 10.1038/s41598-018-34367-x

**Published:** 2018-10-29

**Authors:** Shoji Yokobori, Kevin K. K. Wang, Zhihui Yang, Tian Zhu, Joseph A. Tyndall, Stefania Mondello, Yasushi Shibata, Naoki Tominaga, Takahiro Kanaya, Toru Takiguchi, Yutaka Igarashi, Jun Hagiwara, Ryuta Nakae, Hidetaka Onda, Tomohiko Masuno, Akira Fuse, Hiroyuki Yokota

**Affiliations:** 1 Department of Emergency and Critical Care Medicine, Nippon Medical School, Tokyo, Japan; 2 Program for Neurotrauma, Neuroproteomics & Biomarkers Research, Departments of Psychiatry, University of Florida, Gainesville, Florida USA; 3 Department of Pediatrics, Daping Hospital, Chongqing, Third Military Medical University, No. 10 Changjigang Zhilu, Chongqing, 400042 China; 4 Department of Emergency Medicine, University of Florida, Gainesville, Florida USA; 5 Department of Biomedical and Dental Sciences and Morphofunctional Imaging, University of Messina, Messina, Italy; 6 Oasi Research Institute-IRCCS, Troina, Italy; 7 Department of Clinical Laboratory, Nippon Medical School Hospital, Tokyo, Japan

## Abstract

This study aimed to identify neurological and pathophysiological factors that predicted return of spontaneous circulation (ROSC) among patients with out-of-hospital cardiac arrest (OHCA). This prospective 1-year observational study evaluated patients with cardiogenic OHCA who were admitted to a tertiary medical center, Nippon Medical School Hospital. Physiological and neurological examinations were performed at admission for quantitative infrared pupillometry (measured with NPi-200, NeurOptics, CA, USA), arterial blood gas, and blood chemistry. Simultaneous blood samples were also collected to determine levels of neuron-specific enolase (NSE), S-100b, phosphorylated neurofilament heavy subunit, and interleukin-6. In-hospital standard advanced cardiac life support was performed for 30 minutes.The ROSC (n = 26) and non-ROSC (n = 26) groups were compared, which a revealed significantly higher pupillary light reflex ratio, which was defined as the percent change between maximum pupil diameter before light stimuli and minimum pupil diameter after light stimuli, in the ROSC group (median: 1.3% [interquartile range (IQR): 0.0–2.0%] vs. non-ROSC: (median: 0%), (Cut-off: 0.63%). Furthermore, NSE provided the great sensitivity and specificity for predicting ROSC, with an area under the receiver operating characteristic curve of 0.86, which was created by plotting sensitivity and 1-specificity. Multivariable logistic regression analyses revealed that the independent predictors of ROSC were maximum pupillary diameter (odds ratio: 0.25, 95% confidence interval: 0.07–0.94, P = 0.04) and NSE at admission (odds ratio: 0.96, 95% confidence interval: 0.93–0.99, P = 0.04). Pupillary diameter was also significantly correlated with NSE concentrations (r = 0.31, P = 0.027). Conclusively, the strongest predictors of ROSC among patients with OHCA were accurate pupillary diameter and a neuronal biomarker, NSE. Quantitative pupillometry may help guide the decision to terminate resuscitation in emergency departments using a neuropathological rationale. Further large-scale studies are needed.

## Introduction

The aging population of Japan has created an abrupt increase in the incidence of out-of-hospital cardiac arrest (OHCA). For example, 31,216 patients with OHCA were transferred by emergency medical services (EMS) in 1991, and this number has almost quadrupled to 123,554 patients in 2016, which has created heavy healthcare and economic burdens^1^. To avoid futile treatment, several recommendations for the termination of resuscitation (TOR) have been formulated by healthcare organisations and medical societies and were recently incorporated into the American Heart Association’s cardiopulmonary resuscitation (CPR) guidelines^2,3^. However, these situation-based TOR guidelines, may be strongly affected by regional circumstances and medical systems, for example ability of EMS, quality of bystander CPR, and availability of prehospital automatic external defibrillation (AED). Thus, objective, reliable, and accurate indicators that are rooted in the pathophysiological mechanisms of OHCA are urgently needed to guide TOR decisions^4,5^. This prospective pilot study aimed to identify physiological factors that could guide TOR decisions at the emergency department level.

## Methods

### Study design and setting

This 1-year prospective observational study was performed at an urban tertiary emergency medical center, Nippon Medical School Hospital which has a 60-bed emergency intensive care unit. The study’s protocol was approved by the institutional review board of Nippon Medical School (#27-09-490, Japanese clinical trial identifier: UMIN000015658), and informed consent was obtained from the patients’ relatives or legal representatives. This study was conducted according to the principles of the Declaration of Helsinki, and all methods were performed in accordance with the relevant guidelines and regulations.

The inclusion criteria were patients with cardiogenic or suspected cardiogenic OHCA who were transferred to the ER at our institution. Patients of in-hospital CA or OHCA with evidence return of spontaneous circulation (ROSC) out of hospital were excluded. Patients were also excluded if they had CA that was caused by non-cardiogenic factors (i.e., obvious trauma, asphyxia, sepsis, stroke, drawing, hanging, pulmonary disease, vascular emergency, or terminal cancer), with the causes of CA being defined through the consensus of two board-certified ER physicians. Computed tomography, chest roentgenography, and transthoracic echocardiogram were used to assess the causes of CA. Additional criteria for exclusion were the absence of informed consent, extracorporeal cardiopulmonary resuscitation, and percutaneous cardiopulmonary support in the ER.

Immediately after ER admission, all patients received a standard of 30 minutes ACLS according to American Heart Association CPR guidelines^6^. In-hospital ACLS was continued for 30 minutes and pulsation was checked at the carotid artery every 2 min. Patients were divided into ROSC and non-ROSC groups based on the presence of spontaneous circulation within the 30 min in-hospital standard ACLS. Patients with ROSC received standard care with targeted temperature management (34 °C/48 h) using the Thermogard XP Temperature Management System (ZOLL San Jose, San Jose, CA).

### Quantitative infrared pupillometry

Pupillary diameter and light reflex were measured immediately after the patient’s admission using a quantitative infrared pupilometer (Npi 200; NeurOptics, Irvine, CA)^7^. This device automatically obtains values for maximum pupil diameter (MAX, mm), minimum pupil diameter (MIN, mm), latency of the light reflex (seconds), contraction ratio (%CH = 1 − [MAX − MIN/MAX]), mean contraction velocity (mm/s), and dilation velocity (mm/s)^8^. The measurement was performed twice, and the average was recorded for all parameters.

### Biomarker measurements

Blood samples obtained at admission were centrifuged at 2,500 rpm for 10 min, and the sera were stored at −80 °C until batch biomarker analyses. Commercial sandwich enzyme-linked immunosorbent assay kits were used to detect S100β (EMD Millipore Corporation, Billerica, MA) and phosphorylated neurofilament heavy subunit (BioVendor, Brno, Czech Republic) based on previously described protocols^9^. Detection of NSE was performed using an electrochemiluminescence immunoassay (Roche Diagnostics, Mannheim, Germany).

### Cytokine measurement

Detection of IL-6, which is an inflammatory cytokine, was performed using the Human IL-6 RAYFAST system (RAYFAST, Toray, Tokyo), which provides rapid serum IL-6 measurements^10^. This system measures IL-6 concentrations in biofluids using a fluorescence enzyme immunoassay within 19 min of a 150-µL whole-blood sample being obtained.

### Outcome measurements

The 30-day neurological outcomes in the ROSC group were assessed using the CPC score^11^. Scoring was performed by a single researcher who was blinded to the initial patient data, and good neurological outcomes were defined as CPC scores of 1–2.

### Statistical analysis

Baseline characteristics were reported as number (percentage) or median (interquartile range). Continuous data were compared between the ROSC group and non-ROSC group using the Mann-Whitney U test, while categorical data were compared using the Chi-squared test. The discriminative abilities of the potential biomarkers were evaluated using an AUC. Multivariable logistic regression analysis with the stepwise selection method was used to identify factors that independently predicted ROSC or non-ROSC status. All analyses were performed using StatFlex software (version 6.0; Artech Co. Ltd., Osaka, Japan), and differences were considered statistically significant at P-values of <0.05.

## Results

### Patient enrolment and characteristics

**Figure 1:**
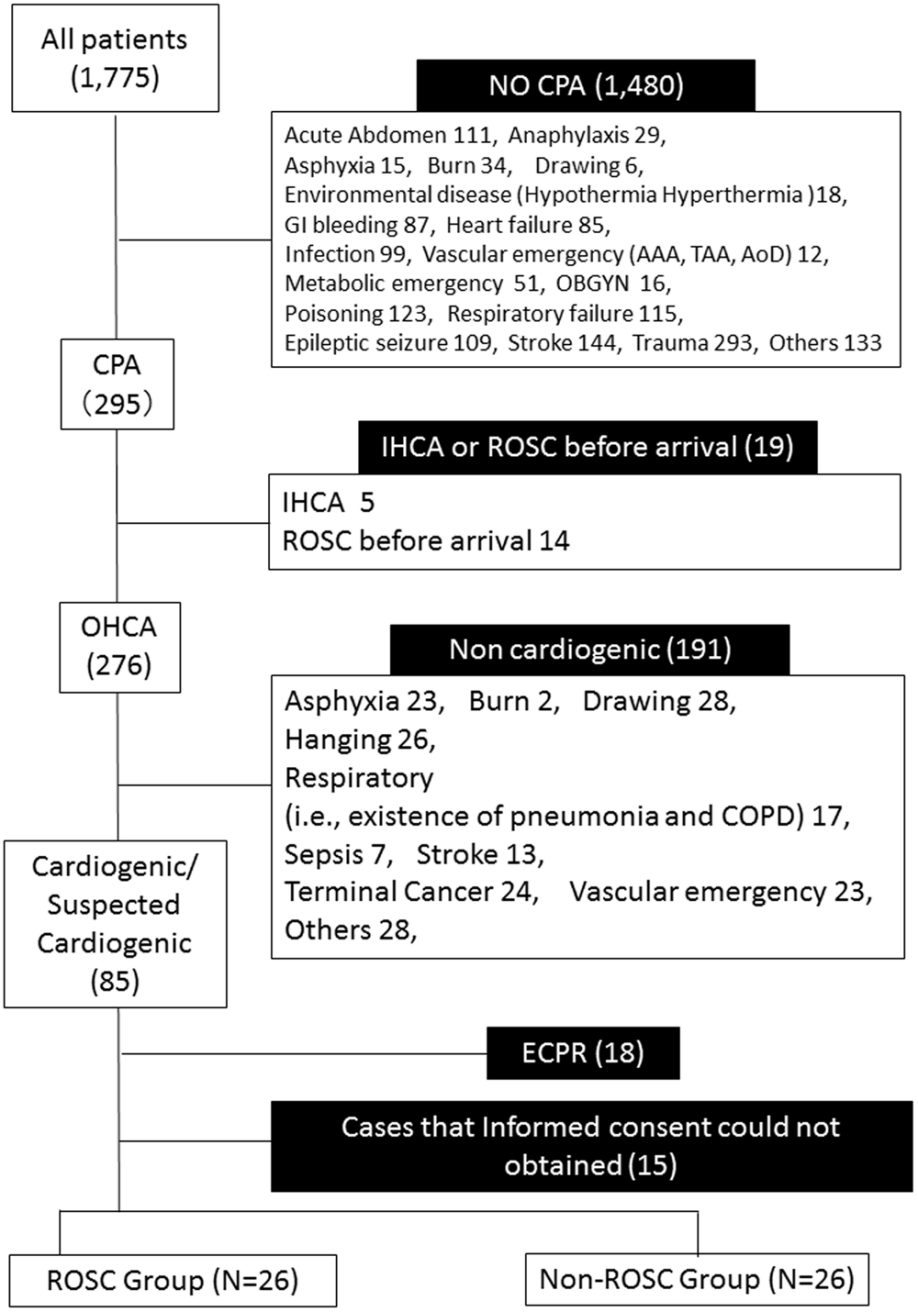
Study flow chart. During the study period, 1,775 patients were admitted to the emergency department, including 276 patients with OHCA. Based on the inclusion and exclusion criteria, a total of 52 patients with OHCA were included in this pilot study. CPA; cardiopulmonary arrest, GI; gastrointestinal, AAA; abdominal aortic aneurysm, TAA; thoracic aortic aneurysm, AoD; aortic dissection, OBGYN; obstetrics and gynaecology, IHCA; in-hospital cardiac arrest, OHCA; out-of-hospital cardiac arrest, COPD; chronic obstructive pulmonary disease, ECPR; extracorporeal cardiopulmonary resuscitation, PCPS; percutaneous cardiopulmonary support, ROSC; return of spontaneous circulation.

Among 1,775 screened cases that were admitted to our institution between January 1 and December 31, 2016, 52 patients were enrolled in the study, including 26 patients with ROSC and 26 patients without ROSC (Fig. 1). Among the eligible patients, 78.8% of the subjects were male, and the median age was 73.0 years (Supplementary Table 1). Of all the enrolled 52 patients, five cases (9.6%) presented with ventricular fibrillation/tachycardia (VF/VT) as the initial rhythm, and 7 patients (13.5%) had received bystander CPR. The median time from the emergency call to the first touch by the EMS team was 8.0 minutes and the median time from the call to hospital arrival was 33.0 minutes. In all enrolled patients, the median 30-day cerebral performance category (CPC) score was 5, and 9.6% of the subjects had good neurological outcomes (CPC 1–2). The ROSC and non-ROSC groups had similar values for their demographic and pre-hospital characteristics, including comorbid medical conditions, pre-hospital treatment, and the times to first EMS touch and hospital admission (Table 1). However, significant differences between the two groups were observed in several initial laboratory findings, including PaO_2_, lactate, potassium, creatine kinase, blood urea nitrogen, creatinine, ammonia, troponin T, and D-dimers (Supplementary Table 2).

**Table 1:** Prehospital characteristics of the ROSC and non-ROSC groups.

	ROSC Group (n = 26) n (%) or median (IQR)	Non-ROSC Group (n = 26) n (%) or median (IQR)	P-value
**Male**	22 (84.6)	19 (73.1)	0.73
**Age, years**	73.0 (66.0–81.0)	72.5 (63.0–82.0)	0.94
**Pre-hospital initial rhythm**			
Asystole	12 (46.2)	18 (69.2)	0.09
PEA	9 (34.6)	6 (23.1)	0.36
VF/VT	5 (19.2)	2 (7.7)	0.22
**Pre-hospital maximum pupil, mm**	5.0 (4.0–6.0)	5.3 (5.0–6.0)	0.51
**Pre-hospital light reflex**	0 (0)	0 (0)	1.0
**Comorbid conditions**			
Hypertension	4 (15.4)	3 (11.5)	0.68
Diabetes mellitus	3 (11.5)	2 (7.7)	0.64
Chronic heart failure	2 (7.7)	4 (15.4)	0.39
Dementia	2 (7.7)	1 (3.8)	0.55
Treated cancer	2 (7.7)	1 (3.8)	0.55
**Witnessed**	14 (53.8)	12 (46.1)	0.57
**Bystander CPR**	11 (42.3)	7 (26.9)	0.24
**Pre-hospital treatment**			
Tracheal intubation	2 (7.7)	2 (7.7)	1.0
Supraglottic device	4 (15.4)	5 (19.2)	0.71
Epinephrine injection	5 (19.2)	4 (15.4)	0.71
Electric defibrillation	6 (23.0)	2 (7.7)	0.12
**Minutes from EMS call to first touch**	8.0 (6.0–10.0)	8.5 (7.0–12.0)	0.36
**Minutes from EMS call to hospital arrival**	32.0 (23.0–37.0)	33.0 (29.0–39.0)	0.22

Data are shown as number (%) or median (interquartile range: IQR).

Abbreviations: IQR; interquartile range, ROSC; return of spontaneous circulation, PEA; pulseless electrical activity, VF/VT; ventricular fibrillation/ ventricular tachycardia, CPR; cardiopulmonary resuscitation, EMS: emergency medical service.

### Pupillometry, serum biomarkers for brain damage, and inflammation

Significant differences between the ROSC and non-ROSC groups were observed for quantitative infrared pupillometry parameters (maximum pupil diameter; MAX and %CH; contraction ratio: Table 2). The non-ROSC patients also had significantly higher serum concentrations of neuron-specific enolase (NSE), S-100b, and interleukin-6 (IL-6) than the ROSC patients, although there was no significant difference in the levels of phosphorylated neurofilament heavy subunit (Table 2).

**Table 2:** Differences in neurological and inflammatory biomarkers.

	ROSC group (n = 26)	Non-ROSC group (n = 26)	P-value
**Brain biomarkers**			
Neuron-specific enolase, ng/mL	24.2 (19.1–44.4)	61.4 (39.4–91.7)	<0.001
S100-β, pg/mL	1562.6 (884.1–2286.6)	2515.7 (1946.9–2999.9)	<0.001
pNF-H, pg/mL	40.7 (0.0–184.4)	35.6 (0.0–118.5)	0.78
**Quantitative infrared pupillometry**			
MAX, mm,	3.9 (3.0–5.0)	5.0 (5.0–6.0)	<0.001
%CH	1.3 (0.0–2.0)	0.0 (0.0–0.0)	<0.001
**Inflammatory cytokine**			
IL-6, pg/mL	42.0 (23.5–144.0)	116.0 (35.0–972.0)	0.04

Data are shown as number (%) or median (interquartile range).

ROSC; return of spontaneous circulation, MAX; maximum pupil diameter, %CH; contraction ratio.

The other pupillometric parameters could not be compared because the light reflex could not be measured in the non-ROSC group.

### Parameters at admission that predicted ROSC

**Figure 2:**
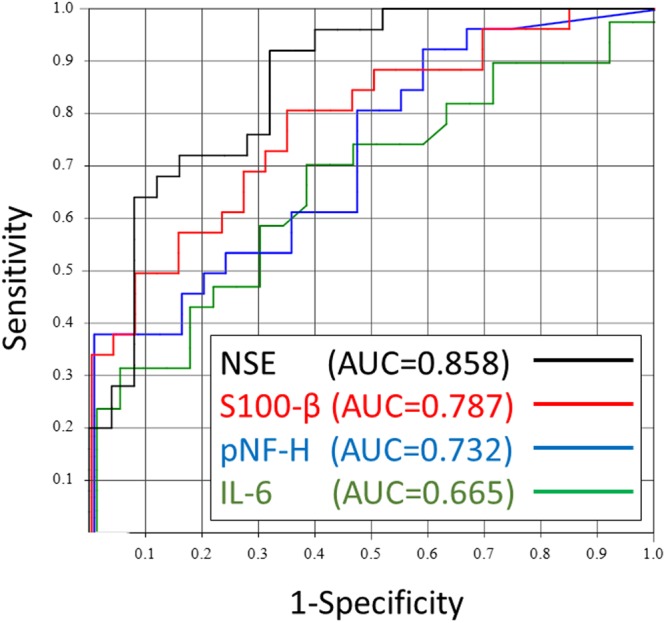
The receiver operating characteristic curve for discriminating between patients who did and patients who did not experience ROSC. The receiver operating characteristic curve analysis was performed using serum brain and inflammatory biomarkers. The strongest predictor of ROSC in the emergency department (ED) was NSE, which provided an area under the curve of 0.858. ROSC; return of spontaneous circulation NSE; neuron-specific enolase, pNF-H; phosphorylated neurofilament heavy subunit, IL-6; interleukin-6, AUC; area under the curve.

Among all neurological and inflammatory biomarkers, the receiver operating characteristic curve analysis revealed that an NSE cut-off value of 40.6 ng/mL provided the greatest area under the receiver operating characteristic curve (AUC) for discriminating between ROSC and non-ROSC cases (AUC = 0.858, Fig. 2). The specific cut-off values of brain biomarkers and pupillometry are listed in Table 3. Multivariable logistic regression analysis revealed that only NSE concentrations and accurate pupillary diameter independently predicted ROSC among patients with OHCA (Table 4).

**Table 3:** Optimal cut-off and area under the curve values for predicting ROSC.

	AUC	Cut-off
**Arterial blood gas**		
PaO2, mmHg	0.786	66.5
Lactate, mg/dL	0.712	118.0
Potassium, mmol/L	0.746	5.5
**Serum chemistry**		
Creatine kinase, U/L	0.697	151.0
Blood urea nitrogen, mg/dL	0.615	18.0
Creatinine, mg/dL	0.654	1.15
NH_3_, µg/dL	0.665	217.75
Troponin T, ng/mL	0.687	0.05
D-dimers, µg/mL	0.689	17.4
**Brain biomarkers**		
Neuron-specific enolase, ng/mL	0.858	40.6
S100-β, pg/mL	0.787	1993.2
**Pupillometry**		
MAX, mm	0.821	4.99
%CH, %	0.731	0.65
**Inflammatory cytokine**		
IL-6, pg/mL	0.665	61.25

ROSC; return of spontaneous circulation, MAX; maximum pupil diameter, %CH; contraction ratio

**Table 4:** Multivariable logistic regression analysis of factors that independently predicted ROSC after OHCA.

	Odds ratio	95% CI	P-value
Neuron-specific enolase	0.96	0.93–0.99	0.041
Maximum pupillary diameter	0.25	0.07–0.94	0.042
PaO_2_	1.02	0.99–1.05	0.111
Potassium	0.53	0.27–1.06	0.073

ROSC; return of spontaneous circulation, OHCA; out-of-hospital cardiac arrest, CI; confidence interval.

### Predictors of 30-day good neurological outcomes

In the ROSC group, 5 patients had good 30-day neurological outcomes, which were defined as CPC 1-2 (Supplementary Table 3). There were no significant differences between the groups with good outcomes (CPC 1–2) and unfavourable outcomes (CPC 3–5) in terms of pre-hospital interventions, existence of a shockable rhythm, and the time to ROSC (Supplementary Table 3). Among the post-admission clinical parameters, a PaO_2_ cut-off value of 248.4 mmHg before the ROSC provided the greatest AUC (0.867), with 80% sensitivity and 80% specificity (Supplementary Table 4). The multivariable logistic regression analysis revealed that the initial in-hospital PaO_2_ value before the ROSC independently predicted good 30-day neurological outcomes (odd ratio: 1.01, 95% confidence interval: 1.00–1.02, P = 0.03) (Supplementary Table 5).

## Discussion

This prospective pilot study aimed to identify objective reliable factors that could predict ROSC among patients with OHCA, and it provides initial evidence that measured quantitative pupillary diameter and serum NSE concentrations at emergency department (ED) admission may be useful in this setting.

In this context, the TOR decision for patients with OHCA remains a major challenge^12^ because of the related medical, legal, and ethical issues. Recent guidelines have provided recommendations and frameworks for guiding the TOR decision, with the National Association of EMS Physicians suggesting that resuscitative efforts could be terminated for patients who have not responded after 20 min of Advanced Cardiac Life Support (ACLS)^13^. A large cohort study from Western countries has also supported a widely accepted TOR decision rule, which recommends considering TOR when all of the following criteria apply before transport: (1) unwitnessed arrest, (2) no bystander CPR, (3) no ROSC after full ACLS in the field, and (4) no AED shocks were delivered^3^. This rule has been validated and adopted for adult patients in several regions of Western countries^14,15^, although the effectiveness of this rule relies heavily on local EMS practices. For example, a report from the Netherlands has indicated that 46% of patients had CPR attempts that were terminated in the field^5^. However, EMS personnel in Japan are not authorised to perform TOR determinations in the field, which must be performed by a physician in the ER. The response time and pre-hospital treatment also vary widely in each region, with Japan still having low rates of highly skilled pre-hospital interventions, such as intubation and adrenaline injection^16,17^.

As the timing of TOR is difficult to decide, a clear pathophysiological rationale is crucial to effectively supporting the TOR decision. For example, established and validated expiratory carbon dioxide cut-off values can be used to predict ROSC after OHCA, although their sensitivity and specificity are suboptimal (sensitivity: 73.9%, specificity: 58.4%, AUC: 63.5)^18^. Thus, there is a clear need for reliable biomarkers that can guide physicians in making the TOR decision. The prognostic abilities of serum NSE and S-100b concentrations from after the ROSC have been evaluated in the post-cardiac arrest setting^19–21^, although there is limited information regarding whether these neurological biomarkers can predict ROSC.

Our findings demonstrate that serum NSE concentrations at the admission independently predicted ROSC with a high AUC value. Furthermore, NSE provided greater sensitivity and specificity than S-100b. In this context, NSE is a 78-kD dimeric γ-isoenzyme of the enolase glycolytic enzyme, and is predominantly localized in the neuronal cytoplasm^22^. In contrast, S-100b is mainly located in the cytoplasm and nucleus of glial cells in the central nervous system^23^. Our results revealed a large difference in the sensitivities of NSE and S-100b, which may be related to the different ischemic tolerances of the specific brain cells^24^. For example, neurons are much more susceptible to ischemia than astrocytes are. These results agree with findings from previous studies of patients with traumatic brain injury, which revealed distinct neuronal and glial biomarker patterns that were associated with specific injury pathways and had important implications for prognostication and therapeutic interventions^25,26^. Thus, our results provide initial evidence that NSE is a stronger predictor of ischemic damage after OHCA than other biomarkers from brain tissues and other organs.

We also found a strong correlation between the concentrations of NSE and D-dimers (r = 0.73, 95% confidence interval: 0.57–0.84, P < 0.001) (Supplementary Table 6). Interestingly, a recent study revealed that concentrations of fibrinogen degeneration product and D-dimers at hospital admission predicted neurological outcomes after OHCA, as increases in D-dimer concentrations were associated with post-arrest anoxia and endothelial injury^27^. Thus, the post-arrest increases in NSE concentrations likely reflect the effects of ischemia and reperfusion. In addition, NSE measurements may be useful for guiding decision-making regarding resuscitation in the ER and in the pre-hospital setting. An assay with a rapid turnaround time would be of tremendous use in these settings, given the compressed timeframes for decision-making after OHCA.

The present study also revealed that pupillary diameter independently predicted ROSC in our study cohort. In this context, the recent development of quantitative infrared pupillometry has enabled rapid and precise measurements of pupillary size and the speed and latency of the pupillary light response (PLR)^6,28^. Behrends *et al*. evaluated 30 patients with cardiac arrest (CA) and detected PLR in 83% of their cohort^9^. They also suggested that the presence of PLR at any point during CPR was associated with early survival and concluded that pupillometry could be used as an indirect marker of brain stem blood flow to guide CPR efforts^9^. In our cohort, pupillary diameter was also significantly correlated with NSE concentrations (r = 0.31, P = 0.027) (Supplementary Table 6), which suggests that pupillary diameter may reflect ischemic damage to neurons in the brain. Therefore, quantitative infrared pupillometry may provide incremental prognostic information to guide the TOR decision, reflecting the pathophysiological impact of cardiac arrest in OHCA patients.

The present study has several limitations. The first major limitation is the small sample size (n = 56), and therefore larger prospective studies are needed to validate the predictive abilities of pupillometry measurements and NSE. Second, this cohort only included patients with cardiogenic or suspected cardiogenic OHCA, and computed tomography was used to exclude cases of neurogenic CA. However, we cannot exclude the possibility that other causes of CA were included in the cohort. Third, resuscitation was performed for 30 min based on our hospital’s standard protocol, although the TOR guidelines recommend stopping resuscitation after 20 min.

In conclusion, this prospective pilot study revealed that initial pupillary diameter and serum NSE concentration may be accurate neurological biomarkers for guiding the TOR decision in the ER setting. Nevertheless, further large-scale studies are warranted to validate these findings.

## Electronic supplementary material

Supplementary Data Set

## Data Availability

Datasets, generated and/or analyzed during the current study, are available from the corresponding author on a reasonable request.
