# Supplementary material for: Quantitative pupillometry and neuron-specific enolase independently predict return of spontaneous circulation following cardiogenic out-of-hospital cardiac arrest: a prospective pilot study
Source: Sci Rep. 2018 Oct 29;8:15964. doi: 10.1038/s41598-018-34367-x (PMC6206016; doi:10.1038/s41598-018-34367-x)
Supplement: Supplementary file 1 — Supplementary Data Set [file 41598_2018_34367_MOESM1_ESM.pdf]

Supplementary Data

Quantitative pupillometry and neuron-specific enolase independently predict return of spontaneous circulation following cardiogenic out-of-hospital cardiac arrest: a prospective pilot study

Shoji Yokobori[a*], Kevin KK Wang[b], Zhihui Yang[b], Tian Zhu[b,c], Joseph A. Tyndall[d], Stefania Mondello[e,f], Yasushi Shibata[a,g], Naoki Tominaga[a], Takahiro Kanaya[a], Toru Takiguchi[a], Yutaka Igarashi[a], Jun Hagiwara[a], Ryuta Nakae[a], Hidetaka Onda[a], Tomohiko Masuno[a], Akira Fuse[a], Hiroyuki Yokota[a]

[a] Department of Emergency and Critical Care Medicine, Nippon Medical School, Tokyo, Japan

[b] Program for Neurotrauma, Neuroproteomics & Biomarkers Research, Departments of Psychiatry, University of Florida, Gainesville, Florida, USA

[C] Department of Pediatrics, Daping Hospital, Chongqing, Third Military Medical University, No. 10 Changjigang Zhilu, Chongqing 400042, China

[d] Department of Emergency Medicine, University of Florida, Gainesville, Florida, USA

[e] Department of Biomedical and Dental Sciences and Morphofunctional Imaging, University of Messina, Messina, Italy

[f] Oasi Research Institute-IRCCS, Troina, Italy

[g] Department of Clinical Laboratory, Nippon Medical School Hospital

Supplementary Table 1. Baseline characteristics of the study cohort.

Parameter	Value
**Number of patients**	52
**Male sex**	41 (78.8)
**Age, years**	73.0 (63.0–81.5)
**Prehospital initial rhythm**	
Asystole	31 (59.6)
PEA	16 (30.8)
VF/VT	5 (9.6)
**Comorbid conditions**	Hypertension: 7 (13.5)<br>Diabetes mellitus: 5 (9.6)<br>Chronic heart failure: 6 (11.5)<br>Dementia: 3 (5.8)<br>Treated cancer: 3 (5.8)
**Witnessed**	15 (28.8)
**Bystander CPR**	7 (13.5)
**Prehospital treatment**	
Tracheal intubation	4 (7.7)

Supraglottic device	9 (17.3)
Adrenaline injection	9 (17.3)
Electric defibrillation	8 (15.4)
**Minutes from EMS call to first touch**	8.0 (6.5–11.0)
**Minutes from EMS call to hospital arrival**	33.0 (27.0–38.5)
**APACHE II score**	41.5 (37.5–45.0)
**30-day outcomes using CPC**	
CPC 1	3 (5.8)
CPC 2	2 (3.8)
CPC 3	2 (3.8)
CPC 4	7 (13.5)
CPC 5	38 (73.1)
**Good 30-day neurological outcomes (CPC 1–2)**	5 (9.6)

Data are shown as number (%) or median (interquartile range).

PEA; pulseless electrical activity, VF/VT; ventricular fibrillation/ventricular tachycardia, CPR; cardiopulmonary resuscitation, EMS: emergency medical service, APACHE; Acute Physiology and Chronic Health Evaluation, CPC; cerebral performance category.

Supplementary Table 2. Physiological data from the admission

	ROSC group (n = 26)	Non-ROSC group (n = 26)	P-value
**Initial rhythm on admission**			
Asystole	12 (46.2)	19 (73.1)	0.05
PEA	12 (46.2)	4 (15.4)	0.03
VF/VT	2 (7.6)	3 (11.5)	0.64
**Body temperature**	36.1 (35.2–36.5)	35.5 (34.9–36.2)	0.21
**Arterial blood gas**			
pH	6.942 (6.836–7.094)	6.873 (6.734–7.015)	0.10
$PaCO_2$, mmHg	93.7 (76.9–106.0)	74.2 (48.0–103.0)	0.07
PaO_2, mmHg	29.5 (16.6–67.1)	109.3 (51.3–285.0)	<0.01
HCO_3, mmol/L	15.2 (12.9–19.6)	15.7 (11.9–20.0)	0.91
Glucose, mg/dL	253 (194–293)	206 (116–304)	0.22
Lactate, mg/dL	106 (77–136)	154 (93–186)	<0.01
Potassium, mmol/L	4.5 (4.0–5.9)	6.4 (5.3–8.5)	<0.01
**Blood cell counts**			
White blood cells, 10^3/μL	10.1 (7.6–13.3)	8.9 (6.8–12.9)	0.37
Haemoglobin, g/dL	12.4 (11.2–14.6)	13.5 (11.4–16.5)	0.31

Platelets, $10^4/\mu L$	18.5 (13.5–21.8)	14.0 (11.7–20.8)	0.27
**Serum chemistry**			
AST, U/L	105 (55–186)	143 (69–550)	0.11
ALT, U/L	67 (29–133)	121 (43–504)	0.05
Creatine kinase, U/L	112 (90–221)	214 (131–473)	0.01
Amylase, U/L	89 (67–130)	118 (90–192)	0.05
Total bilirubin, mg/dL	0.5 (0.3–0.6)	0.5 (0.3–0.7)	0.34
Blood urea nitrogen, mg/dL	16.0 (12.3–25.5)	24.4 (16.6–37.2)	0.02
Creatinine, mg/dL	1.1 (0.8–1.2)	1.3 (1.1–2.0)	0.02
Total protein, g/dL	6.2 (5.6–6.6)	6.6 (6.2–7.4)	0.05
Albumin, g/dL	3.3 (2.8–3.7)	3.2 (2.8–4.0)	0.75
C-reactive protein, mg/dL	0.2 (0.0–1.6)	0.5 (0.2–4.9)	0.08
Procalcitonin, ng/mL	0.1 (0.0–0.1)	0.1 (0.0–0.4)	0.27
NH_3, μg/dL	191 (104–283)	274 (157–469)	0.04
Troponin T, ng/mL	0.0 (0.0–0.1)	0.1 (0.0–0.2)	0.03
D-dimers, μg/mL	15.6 (4.2–49.3)	48.4 (14.7–183.6)	0.02

Data are shown as n (%) or median (interquartile range).

PEA; pulseless electrical activity, VF/VT; ventricular fibrillation/ventricular tachycardia, CPR; cardiopulmonary resuscitation, AST; aspartate transaminase, ALT; alanine transaminase.

Supplementary Table 3. Clinical parameters predicting 30-day neurological outcomes

	Good (CPC 1–2)	Unfavourable (CPC 3–5)	P-value
**Number of patients**	5	21	-
**Male sex**	5 (100)	17 (77.3)	0.268
**Age, years**	71.0 (54.5–79.0)	73.0 (67.5–81.3)	0.696
**Minutes from EMS call to ROSC**	26.0 (18.5–37.3)	36.5 (30.0–55.5)	0.208
**Prehospital initial rhythm**			
Asystole	1 (20.0)	11 (52.4)	0.192
PEA	2 (20.0)	7 (33.3)	0.778
VF/VT	2 (20.0)	3 (14.3)	0.190
**Pre-hospital maximum pupillary diameter, mm**	5.0 (4.0–6.0)	6.0 (3.5–5.0)	0.910
**Pre-hospital light reflex** (%)	0 (0)	0 (0)	1.000
**Comorbid conditions**			
Hypertension	0 (0)	3 (14.3)	0.369
Diabetes mellitus	0 (0)	3 (14.3)	0.369
Chronic heart failure	0 (0)	2 (9.5)	0.473
Dementia	1 (20.0)	1 (4.8)	0.251
Treated cancer	0 (0)	2 (9.5)	0.473

**Witnessed**	4 (80.0)	10 (47.6)	0.192
**Bystander CPR**	3 (60.0)	8 (38.1)	0.372
**Pre-hospital treatment**			
Tracheal intubation	0 (0)	2 (9.5)	0.473
Supraglottic device	1 (20.0)	3 (14.3)	0.750
Epinephrine injection	0 (0)	5 (23.8)	0.225
Electric defibrillation	2 (40.0)	4 (19.0)	0.318
**Minutes from EMS call to first touch**	9.0 (6.3–9.3)	8.0 (6.0–10.5)	0.844
**Minutes from EMS call to hospital arrival**	28.0 (25.5–34.8)	33.0 (23.0–37.0)	0.648
**Initial rhythm on admission**			
Asystole	1 (20.0)	11 (52.4)	0.192
PEA	2 (40.0)	10 (47.6)	0.759
VF/VT	2 (40.0)	0 (0)	0.026
**Body temperature, ºC**	36.5 (36.4–36.6)	35.6 (35.0–36.5)	0.051
**Arterial blood gas**			
pH	7.047 (6.842–7.323)	6.922 (6.831–7.039)	0.283
$PaCO_2$, mmHg	34.4 (33.8–134.3)	77.3 (58.4–100.3)	0.626
PaO_2, mmHg	376.0 (221.2–514.5)	76.4 (44.4–233.0)	0.012

HCO_3, mmol/L	19.6 (14.4–21.1)	14.2 (12.8–17.7)	0.329
Glucose, mg/dL	290.0 (264.5–301.1)	249.0 (179.8–284.8)	0.126
Lactate, mg/dL	92.0 (69.3–131.5)	110.0 (81.8–136.5)	0.696
Potassium, mmol/L	3.7 (3.2–4.6)	4.7 (4.2–6.1)	0.047
**Blood cell counts**			
White blood cells, $10^3/\mu L$	8,000 (7,250–9,725)	11,400 (7,770–14,925)	0.152
Haemoglobin, g/dL	14.6 (13.3–15.5)	12.2 (11.0–13.3)	0.162
Platelets, $10^4/\mu L$	19.5 (17.0–23.6)	18.4 (12.4–21.7)	0.380
**Serum chemistry**			
AST, U/L	36.0 (35.3–83.0)	122.0 (61.0–196.3)	0.044
ALT, U/L	29.0 (21.5–56.0)	80.0 (37.0–139.8)	0.085
Creatine kinase, U/L	110.0 (90.8–136.3)	115.0 (87.8–288.5)	0.362
Amylase, U/L	92.0 (78.5–167.8)	80.0 (65.8–135.8)	0.696
Total bilirubin, mg/dL	0.5 (0.3–0.6)	0.4 (0.3–0.5)	0.551
Blood urea nitrogen, mg/dL	10.7 (8.9–13.7)	16.6 (13.8–30.5)	0.015
Creatinine, mg/dL	0.9 (0.9–1.0)	1.1 (0.8–1.3)	0.182
Total protein, g/dL	6.3 (5.9–6.6)	6.2 (5.6–6.7)	0.896
Albumin, g/dL	3.7 (3.4–3.9)	3.0 (2.8–3.6)	0.067
C-reactive protein, mg/dL	0.04 (0.03–0.13)	0.45 (0.07–2.38)	0.054

Procalcitonin, ng/mL	0.0 (0.0–0.1)	0.1 (0.0–0.1)	0.288
NH_3, μg/dL	168 (73–265)	194 (114–285)	0.500
Troponin T, ng/mL	0.0 (0.0–0.1)	0.0 (0.0–0.1)	0.546
D-dimers, μg/mL	12.0 (7.6–17.8)	16.2 (4.2–60.9)	0.313
**Brain biomarkers**			
Neuron-specific enolase, ng/mL	20.8 (19.3–22.0)	27.4 (19.0–45.5)	0.248
S100-β, pg/mL	842.5 (378.6–2,063.5)	1,665.0 (1,212.5–2,307.0)	0.345
pNF-H, pg/mL	0.0 (0.0–237.1)	52.0 (4.2–188.9)	0.412
**Infrared pupillometry**			
MAX, mm	3.0 (2.9–3.4)	4.0 (3.0–5.0)	0.141
%CH	2.0 (0.8–4.0)	0.0 (0.0–1.0)	0.087
**Inflammatory cytokine**			
IL-6, pg/mL	22.0 (14.0–51.3)	48.0 (28.8–209.5)	0.048

Data are shown as number (%) or median (interquartile range).

PEA; pulseless electrical activity, VF/VT; ventricular fibrillation/ventricular tachycardia, CPR; cardiopulmonary resuscitation, EMS; emergency medical services, AST; aspartate transaminase, ALT; alanine transaminase, MAX; maximum pupil diameter, %CH: contraction ratio.

Supplementary Table 4. Optimal cut-off values and areas under the curves for detecting good 30-day neurological outcomes (CPC1–2).

	Area under the curve	Cut-off
**Arterial blood gas**		
PaO_2, mmHg	0.867	248.4
Potassium, mmol/L	0.819	4.12
**Serum chemistry**		
BUN, mg/dL	0.857	13.1
AST, U/L	0.793	65.3
**Brain biomarkers**		
NSE, ng/mL	0.670	20.5
S-100b, pg/mL	0.638	1431.0
pNF-H, pg/mL	0.676	19.2
**Pupillometry**		
MAX, mm	0.714	2.94
%CH, %	0.728	1.46
**Inflammatory cytokine**		
IL-6, pg/mL	0.78	36.8

BUN; blood urea nitrogen, AST; aspartate transaminase, NSE; neuron-specific enolase, MAX; maximum pupil diameter, %CH; contraction ratio.

Supplementary Table 5. Multivariable logistic regression analysis for independent predictors of good 30-day neurological outcomes (CPC1–2).

Parameter	Odds ratio	95% CI	P-value
PaO_2	1.01	1.00-1.02	0.0346
AST	0.97	0.93-10.1	0.1449

CI: confidence interval, AST; aspartate transaminase.

Supplementary table 6. Linear regression analysis of comparisons with the NSE values.

**Parameter compared with NSE**	**Correlation coefficient (r)**	**95% CI**	**P-value**
D-dimer	0.73	0.57, 0.84	<0.001
Body temperature	-0.59	–0.75, –0.38	<0.001
$PaCO_2$	0.42	0.16, 0.63	0.002
S-100 β	0.32	0.04, 0.55	0.025
Maximum pupillary diameter	0.31	0.04, 0.54	0.027

NSE; neuron-specific enolase, CI; confidence interval.
